# Coaches’ adoption and implementation of the South African long-term athlete development model in cricket

**DOI:** 10.17159/2078-516X/2025/v37i1a19806

**Published:** 2025-12-15

**Authors:** KL Mokwena, A Kubayi

**Affiliations:** Department of Sport, Rehabilitation and Dental Sciences, Tshwane University of Technology, Pretoria, South Africa

**Keywords:** Coaching, winning, performance, player development, stages of development

## Abstract

**Background:**

Although Cricket South Africa has implemented the Long-Term Athlete Development (LTAD) model, there is limited information available about the adoption and implementation of its principles among cricket coaches.

**Objectives:**

This study aimed to gain a better understanding of how South African cricket coaches adopt and implement the LTAD model.

**Methods:**

This study used a quantitative design approach. The sample comprised 86 cricket coaches from nine provinces in South Africa. Coaches responded to questions on a 5-point Likert scale. Data are reported as the mean ± standard deviation.

**Results:**

Overall, coaches reported that the LTAD model helped them to improve their players’ performance (4.33±0.90), contributed to their players’ development (4.31±0.86), and could be adapted to their coaching needs (4.19±0.87). Also, the LTAD could be effective for the benefit of their players (4.16±0.93) and had agreeable theoretical principles (4.02±0.92). Coaches indicated that the barriers to adopting the LTAD model were the need to educate new coaches and parents (4.33±0.84) and the need to better understand the model’s general principles, associated science, and coaching (4.29±0.78). Other barriers included the model’s incompatibility with an emphasis on results and competition (3.79±1.08), as well as the players’ parents’ desire to win at all costs (3.58±1.26).

**Conclusion:**

These results have implications that can be used to provide recommendations which may help Cricket South Africa promote the LTAD model in the South African context.

The primary role of a sports coach is to develop the psychological, social, physical and technical skills of athletes to help them achieve their goals.^[1]^ Reaching a high level in competitive sport usually requires lengthy preparation that may take several years and should be thoroughly periodised.^[2,3]^ Well-structured periodisation for practice and competition can help ensure optimal performance and development throughout an athlete’s career.^[4]^ Numerous models have been developed to help coaches train players to achieve their full potential.^[5,6]^ One such model, which has been widely adopted in sports such as athletics, baseball, gymnastics, rugby and soccer in countries such as Australia and Canada, is the long-term athlete development (LTAD) model devised by Dr Istvan Balyi.^[5–7]^

The LTAD model is based on the principles of development and growth, as well as skills acquisition.^[5,8]^ It consists of the following seven stages of development: (1) Active Start – ages 0 to 6; (2) Fundamentals – ages 6 to 9; (3) Learn to Train – ages 9 to 13; (4) Train to Train – ages 13 to 15 for girls and 13 to 16 for boys; (5) Train to Compete – ages 15 to ±21 for females and 16 to ±23 for males; (6) Train to Win – ages 18+ for females and 19+ for males; and (7) Active for Life – any age.^[9]^ For example, the first three stages (Active Start, Fundamentals, and Learn to Train) serve as the cornerstones for developing participation-and performance-oriented sports. The next three stages (i.e. Train to Train, Train to Compete and Train to Win) are all centred on excellence and the pursuit of high-performance training and competition.^[9]^ The LTAD model was chosen over other models because it provides a framework for young athletes to enjoy sport through structured play while they gradually learn the sport-specific skills needed to compete at the highest level.^[5]^

The LTAD model is introduced to coaches, in both its generic and sports-specific versions, through coaching education programmes.^[5]^ The LTAD model was introduced to South African cricket in 2005 as part of the Coaches Academy review process. The LTAD model adopted from the England and Wales Cricket Board was included in all Cricket South Africa (CSA) coaching education material and formed the foundation of CSA’s coaching philosophy.^[9]^ In 2011, CSA took the logical next step by aligning itself with the South African Sports Confederation and Olympic Committee’s Long-Term Player Development method and creating a more complete development framework based on the Canadian Sport for Life LTAD model.^[9]^ The LTAD model serves as a tool to assist new and already certified coaches in understanding coaching concepts, and it is one of the primary sources of knowledge available to cricket coaches.^[5,10]^ The LTAD model is a recognised pathway for cricket coaches to develop their athletes and increase their participation in physical literacy.^[11]^ Therefore, it is important to understand how coaches perceive the LTAD model and what motivates them to adopt or reject it.^[5]^

To date, few studies have investigated coaches’ adoption and implementation of the LTAD model. For instance, Lang and Light^[8]^ investigated the perceptions of UK swimming coaches regarding how they interpreted and implemented the LTAD model and found that they had concerns about the LTAD model’s overemphasis on training volume at the expense of precise technique. Beaudoin et al.^[5]^ explored barriers to the adoption and implementation of Sport Canada’s LTAD model among coaches of various sports (e.g. athletics, baseball, gymnastics, soccer). The identified barriers included a lack of organisational support for implementing the model, a shortage of evidence-based research on the model, and the complexity of the model when viewed in its entirety.

In Portugal, Costa et al. ^[12]^ examined the relationship between swimming coaches’ experience and their views on the implementation of the LTAD model. The results showed that 95% of coaches accepted the model, and 83% of coaches had confidence in its usefulness. The findings also demonstrated a correlation between experience and understanding of the model, with more experienced coaches demonstrating greater understanding than their less experienced counterparts.^[12]^ The experience of coaches seems to be the main underlying factor influencing athletes’ development.^[13]^ Therefore, coaches’ experience may impact how they learn about, interpret and apply the LTAD model developed by national sports federations.^[12]^

Although the LTAD model has been implemented in South African cricket,^[9]^ there is limited information about the adoption and implementation of its principles among cricket coaches in South Africa. Research is required to help understand the challenges to coaches’ adoption of the LTAD model and how they perceive the model within the context of their sport.^[2,10]^ This research may help policymakers and practitioners to approach the adoption and implementation of talent development in general, and the LTAD model more specifically, from the perspective of sports coaches.^[5]^ In this regard, this study aimed to investigate how cricket coaches adopt and implement the LTAD model and to understand better the barriers they encounter based on their coaching experience in South Africa.

## Methods

### Research design and participants

A cross-sectional research survey was adopted in this study. The sample consisted of 86 cricket coaches (Age = 36.3±8.0 years) in several South African provinces.

### Questionnaire

A questionnaire adapted from a previous study ^[14]^ was used to collect data. The questionnaire solicited information relating to coaches’ understanding of the LTAD model and the perceived barriers to its adoption and implementation. The items were anchored differently based on the type of question. For example, the coaches’ perceptions of adopting and implementing the LTAD model were anchored on a 5-point Likert scale ranging from 1 (*not at all*) to 5 (*very much*). The barriers to cricket coaches’ adoption and implementation of the LTAD model were anchored on a 5-point Likert scale ranging from 1 (*strongly disagree*) to 5 (*strongly agree*). The overall Cronbach’s alpha coefficient for the questionnaire was 0.77, demonstrating that the questionnaire was reliable.

### Data collection procedure

Before data collection, the research was submitted to the Tshwane University of Technology’s Faculty of Science Research Ethics Committee for approval. The participants were recruited through CSA. An information leaflet was sent to cricket coaches to invite them to participate in the study. Data were collected electronically using SurveyMonkey. Participants were required to sign an online informed consent form before participating in the study. Ethical considerations such as confidentiality and privacy were addressed, and questionnaire responses were anonymised to prevent the identification of individual participants.

### Data analysis

Frequency counts and percentages were used to analyse the demographic data of the coaches. The data related to the coaches’ perceptions of the LTAD model and the barriers to its adoption and implementation were presented as mean±standard deviation (SD). An independent *t*-test was applied to examine differences between experienced and less experienced coaches. Cronbach’s alpha coefficients were computed to determine the internal consistency of the questionnaire. The significance level was set at ≤0.05. All statistical analyses were computed using IBM SPSS Statistics (version 25).

## Results

Fourteen cricket coaches were female (16%) and 72 were male (84%). In line with previous research ^[12]^, coaches’ experience was categorised as follows: novice/intermediate coaches (0–9 years, n=34 coaches) and experienced coaches (10+ years; n=52 coaches). The most common coaching qualifications were level 1 (22%), level 2 (58%) and level 3 (15%), while the least common was level 0 (5%). Most coaches worked at the provincial level (55%), followed by hubs/regional performance centres (34%) and franchise (6%) levels. Most of the coaches were from Gauteng (28%), the Eastern Cape (20%), Limpopo (20%) and KwaZulu-Natal (12%). Few of the coaches were from Mpumalanga (6%), the Free State (5%), the Western Cape (2%) and the Northern Cape (1%). Seven per cent of the coaches did not indicate where they were from.

Table 1 shows coaches’ perceptions of the LTAD model based on their coaching experience.^[1]^ The results demonstrated that the LTAD model helped coaches to improve their players’ performance (4.33±0.90), contributed to players’ development (4.31±0.86), could be adapted to coaches’ coaching needs (4.19±0.87), could be effective for or benefit players (4.16±0.93) and had agreeable theoretical principles (4.02±0.92). Significant differences (*p*<0.05) were observed in novice/intermediate coaches’ and experienced coaches’ responses to whether the LTAD model helped to improve players’ performance and whether the ideas proposed by the LTAD model were compatible with their clubs’ or sporting associations’ operating standards and regulations.

Table 2 illustrates the barriers to the adoption and implementation of the LTAD model, as perceived by coaches based on their coaching experience. Coaches indicated that the barriers to adopting the LTAD model were the need to educate new coaches and parents (4.33±0.84); the need to better understand the model’s general principles, associated science and coaching (4.29±0.78); the model’s incompatibility with an emphasis on results and competition (3.79±1.08); and players’ parents’ desire to win at all costs (3.58±1.26). The barriers to implementing the LTAD model identified by coaches were a lack of organisational support (3.77±1.11) and a lack of financial resources (3.74±1.33). Novice/intermediate coaches differed significantly (*p*<0.05) from experienced coaches regarding whether limited information or poor understanding of various stages of development detailed in the LTAD model impeded their adoption of the model.

## Discussion

This study examined how cricket coaches adopt and implement the LTAD model in South Africa. This is the first study of its kind in South Africa to focus on LTAD since its implementation 20 years ago. Overall, the coaches agreed with the theoretical principles of training proposed in the LTAD model and reported a variety of benefits associated with its adoption. It helped them improve their players’ performance, contributed to their players’ development, and could be adapted to meet their coaching needs, ultimately benefiting their players. These results demonstrate that South African cricket coaches value the LTAD model’s contribution to holistic player development. Coach education programmes may be able to foster a constructive and positive attitude to the adoption and implementation of the LTAD model ^[10]^. However, this was not addressed in the current study. Such programmes have a clear role in showing coaches the benefits of the LTAD model and how it aligns with their own beliefs and the requirements of their players.^[14]^

The findings showed significant differences between the responses of novice/intermediate coaches and those of their more experienced counterparts regarding whether the LTAD model helped to improve players’ performance and whether the ideas proposed by the model were compatible with their clubs’ or sporting associations’ operating standards and regulations. Experienced coaches generally held more positive views about the model than novice/intermediate coaches. It could be argued that coaches with more experience are better equipped to look for information that could support their training programmes.^[12]^

Coaches indicated that the most significant barrier to adopting the LTAD model was the need to educate new coaches and parents. It has previously been reported that parents lacked knowledge and understanding regarding the specific recommendations and suggested training sequence of the LTAD model.^[15]^ According to Balyi et al. ^[7]^, parents should exercise patience when their children are going through puberty, as there is a chance that growth spurts could impede the acquisition of skills. Parents should be made aware of this so they can better support the athletes and help them cope with learning plateaus and associated frustrations.^[14]^

Coaches indicated that the LTAD model appears to prioritise team results, with a focus on achieving victory at all costs. It is essential to note that while an early adoption of a winning-at-all-costs mindset in young players’ careers can yield short-term gains, it does not foster long-term involvement in sports.^[7]^ In fact, it has been said that youth teams should primarily seek to develop good individual athletes, creating a positive experience and enjoyment rather than just winning teams.^[15]^ Coaches may be better equipped and more willing to adhere to LTAD principles if the ‘win at all costs’ paradigm is altered. Sports and education administrators may also promote the use of the LTAD model by incorporating it into their schools’ physical education and health curricula.^[2]^

A lack of organisational support was highlighted as a barrier to implementing the LTAD model, as previously reported in other South African studies. ^[16,17]^ Another barrier coaches reported was the allocation of insufficient financial resources by the association to help implement the LTAD model. This result is in line with that of Beaudoin et al.,^[5]^ who noted that Canadian coaches believed that short-term results were still the primary focus of funding, which posed the biggest obstacle to the adoption of the LTAD model in their particular sport. Therefore, to promote and encourage the adoption and implementation of the LTAD model across the country, CSA should provide financial assistance to coaches so they may become familiarised with the model.

Furthermore, significant differences were found regarding a lack of information and understanding about the adoption of the model, with experienced coaches reporting greater barriers than their novice/intermediate counterparts. Given their extensive coaching backgrounds, experienced coaches might have a deeper comprehension of the model than their less experienced counterparts. This could have led to the experienced coaches identifying areas in the model that require attention. Despite the significant effort and resources invested by CSA to develop the model 20 years ago, it has not been researched since its implementation. This is especially significant in the South African context, as the model’s implementation does not imply that it has gained widespread acceptance among stakeholders.

### Practical implications

The study highlighted several ways for CSA to enhance the adoption and implementation of the LTAD model by cricket coaches. Not surprisingly, the coaches highlighted that additional CSA funding was required to adopt and implement the LTAD adequately. This is especially important at the youth club operations level. Additionally, allocating additional funds would aid in standardising the curriculum and content presented in CSA coaching courses and improve consistency in LTAD implementation across clubs. This could help coaches concentrate on promoting the holistic development of athletes and educating parents instead of emphasising winning at all costs, which could lead to youngsters quitting cricket at an early age.

### Limitations and future research

This study features several noteworthy limitations that merit careful consideration. The small sample size stands out as a significant constraint. Additionally, the nationwide distribution of the study encountered discrepancies in response rates among different provinces. For example, almost 80% of the cricket coaches were from Gauteng, the Eastern Cape, Limpopo and KwaZulu-Natal, which limits the generalisability of the results to the other five provinces in South Africa. This variability in engagement rates introduces a potential source of bias, as certain regions may be overrepresented or underrepresented. Additionally, cricket coaches were not asked if they were familiar enough with the LTAD model to answer the questions effectively. Such a lack of understanding could have skewed the responses from the participants. Although this study provides valuable insights into coaches’ perceptions of the LTAD model, further research is needed in this area. Therefore, the principles of South Africa’s LTAD model in cricket should be assessed and informed by ongoing research. Future research should adopt a mixed-methods approach and include other stakeholders who are integral to the LTAD model and its success.

## Conclusion

The findings highlighted that the LTAD model reportedly helped cricket coaches enhance and develop their players’ performance. Thus, this research suggests that the South African LTAD model for cricket is a viable paradigm that coaches should adopt and utilise, as it has practical implications for players’ development pathways. Furthermore, coaches emphasised the need to educate new coaches and parents about the LTAD model; the need to gain a deeper understanding of the model’s general principles, related science and coaching; the incompatibility of the model with a focus on competition and results; and players’ parents’ relentless pursuit of winning at all costs as barriers to adopting the model.

## Figures and Tables

**Table 1:** Novice/intermediate and experienced coaches’ responses to LTAD questions

Questions on LTAD	All n=86	N/IC n=34	EC n=52	p-value
Currently, with the knowledge of LTAD that you have, do you feel capable of implementing most of its principles in your training plans?	3.86±1.00	3.69±0.95	4.00±1.02	0.18
Do you think that LTAD can help to improve the performance of your players?	4.33±0.90	4.08±1.09	4.53±0.65	0.02*
Do you agree with the theoretical principles proposed in the LTAD document?*	4.02±0.92	3.87±1.00	4.15±0.83	0.17
Do you think that LTAD could be effective for or benefit your players?	4.16±0.93	4.05±0.97	4.26±0.90	0.31
Do you think that LTAD may contribute to the development of your players?	4.31±0.86	4.18±0.85	4.43±0.85	0.19
Do you believe that LTAD implementation could potentially limit your ability to act independently as a coach	3.13±1.30	3.08±1.33	3.17±1.29	0.74
Do you think that it is possible to adapt LTAD to your coaching needs?	4.19±0.87	4.05±0.92	4.30±0.83	0.19
Are the ideas proposed by the LTAD model easily compatible (in accordance) with the operating standards and regulations of your club or sports association?	3.78±0.95	3.56±1.07	3.96±0.81	0.05*
In your opinion, is LTAD viewed favorably by the members (coaches, athletes or technical directors) of your sports discipline?	3.77±1.06	3.54±1.07	3.96±1.02	0.07
In your opinion, do the coaches involved in your sport know the rationale, principles, and objectives of LTAD?	3.28±1.18	3.28±1.17	3.28±1.21	0.98
Do you believe that the federation in charge of coaching education for your sport helps you enough to understand and adopt LTAD?	3.30±1.29	3.21±1.17	3.38±1.39	0.53

Data are expressed as mean ± standard deviation.

* Significant at p<0.05.

N/IC, Novice/intermediate coaches; EC, Experienced coaches. Rating scale: 1–5

**Table 2:** Barriers to cricket coaches’ adoption and implementation of LTAD model

Barriers to LTAD adoption	All n=86	N/IC n=34	EC n=52	p-value
Lack of LTAD knowledge and training	3.18±1.08	3.34±1.02	3.04 ±.11	0.21
Emphasis placed on winning at all cost	3.38±1.17	3.34±1.21	3.41±1.15	0.78
Coaches and parents who want to win at all costs	3.58±1.26	3.47±1.13	3.67±1.37	0.47
It goes against long-term vision	3.45±1.35	3.37±1.36	3.52±1.35	0.61
Need for education of new coaches and parents	4.33±0.84	4.45±0.76	4.24±0.90	0.26
Need to better understand the general principles of LTAD, associated science and coaching	4.29±0.78	4.29±0.73	4.28±0.83	0.97
Emphasis on results and competition works against LTAD	3.79±1.08	3.79±1.04	3.78±1.11	0.98
**Barriers to implementation of LTAD**				
Lack of information on some stages of development in the LTAD model	3.46±1.08	3.21±1.09	3.68±1.03	0.05*
Lack of understanding of the other stages of development within the model	3.49±1.10	3.21±1.04	3.72±1.11	0.03*
Lack of financial resources	3.74±1.33	3.76±1.34	3.73±1.34	0.90
Lack of support offered by the organisation	3.77±1.11	3.66±1.07	3.86±1.15	0.41
No technical and tactical drills that give coaches the tools to implement the LTAD model	3.33±1.13	3.29±1.25	3.36±1.04	0.77
Insufficient interaction between members of the organisation	3.52±1.04	3.63±1.08	3.43±1.02	0.39
It is hard to implement LTAD model directions explicitly for certain stages of development	3.44±1.11	3.50±1.20	3.39±1.04	0.65

Data are expressed as mean ± standard deviation.

* Significant at p<0.05.

N/IC, Novice/intermediate coaches; EC, Experienced coaches. Rating scale: 1–5
